# PP35 Assessing Medical Devices: A Qualitative Study From The Perspective Of “Values In Doing Assessments Of Health Technologies”

**DOI:** 10.1017/S0266462325102547

**Published:** 2025-12-29

**Authors:** Bart Bloemen, Zsófia Türk, Wija Oortwijn

## Abstract

**Introduction:**

To identify challenges in adopting new methodologies for assessing medical devices we aimed to explore underlying views of health technology assessment (HTA) practitioners about appropriate methodology at HTA agencies. Inspired by the “values in doing assessments of health technologies” (VALIDATE) approach, we focused on the role of normative commitments of HTA practitioners in the adoption of new methods.

**Methods:**

Based on desk research, an online survey (including questions on procedures, scoping, and assessments of high risk medical devices) was developed and sent to members of the International Network of Agencies for Health Technology Assessment. Semi-structured interviews were conducted with survey respondents involved in assessments of transcatheter aortic valve implantation to gain an in-depth understanding of choices made and views about assessing medical devices. Data collection occurred between January and May 2023. Descriptive statistics were used to summarize survey findings, thematic analysis for the interviews, and the Consolidated Criteria for Reporting Qualitative Research checklist was used for reporting.

**Results:**

Twenty-two out of 50 survey invitees responded, and 15 indicated they assessed medical devices. Among these, eight were willing to be interviewed, of which four accepted the interview invitation. The consolidated results showed that the current practice of assessing medical devices at HTA agencies is predominantly based on procedures, methods, and epistemological principles developed for assessments of drugs. Both practical factors (available time, demands of decision-makers, existing legal frameworks, and HTA guidelines), as well as the commitment of HTA practitioners to principles of evidence-based medicine, made the adoption of new HTA methodology difficult.

**Conclusions:**

There is broad recognition that assessments of medical devices may require changes in HTA methodology. To realize this, the HTA community may need to discuss the role, responsibility, and goals of HTA, and to inform decisions on how to implement new HTA processes and methods for assessing devices.

